# 2753. Real-World Use of Cefiderocol Treating Non-COVID Patients with Confirmed Gram-Negative Infections in US Hospitals During January 2020-June 2022

**DOI:** 10.1093/ofid/ofad500.2364

**Published:** 2023-11-27

**Authors:** Bin Cai, Yun Zhou, Christine M Slover, Jennifer D Copeland, Sean T Nguyen

**Affiliations:** Shionogi Inc, Florham Park, New Jersey; Genesis Research Inc, Hoboken, New Jersey; Shionogi Inc., Florham Park, New Jersey; Shionogi, Inc., Houston, Texas; Shionogi Inc., Florham Park, New Jersey

## Abstract

**Background:**

Cefiderocol (CFDC) has broad activity against Gram-negative (GN) pathogens. This study describes the CFDC usage in treating non-COVID patients with microbiology data in US hospitals from Jan 2020-Jun 2022.

**Methods:**

This retrospective multicenter observational study included adult non-COVID hospitalized patients treated with CFDC consecutively for ≥3 days after laboratory confirmed GN infections between Jan 2020 to Jun 2022, as captured by Premier Healthcare data. This study describes the patient characteristics, microbiological profile, and overall, as well as 14-day and 28-days, in-hospital all-cause mortality (IHACM). Index culture was defined as the first clinical culture with GN pathogens. Index pathogens were all pathogens identified from the index culture.

**Results:**

Patient characteristics of 275 hospitalized patients who received ≥3 days CFDC after the microbiological results are shown in Table 1. For index culture, 61.1% were taken ≤3 days of admission, 53.1% were taken during ICU stay, 45.8% involved respiratory culture, followed by urinary (19.6%), wound (18.2%) and blood (16.4%). Also 65.8% of patients had one index pathogen and 34.2% had ≥2 index pathogens. The most common index pathogens were *Pseudomonas aeruginosa* (48.7%), *Acinetobacter baumannii* (23.6%), *Klebsiella pneumoniae* (14.2%), and *Stenotrophomonas maltophilia* (13.1%). On the index culture day, 62.2% of patients had one pathogen from a single site and 37.8% had either one pathogen identified in multiple culture sites or multiple pathogens from ≥1 culture site.

CFDC was used as monotherapy in 92 (33%) patients, though 253 (92%) had used other GN antibiotics before starting CFDC. A total of 135 (49.1%) patients started CFDC ≤ 5 days of index culture, 108 (39.3%) patients between 6-20 days, and 32 (11.6%) patients > 20 days.

Crude overall IHACM was 16.4% (95%CI:12.0%-20.7%): 10.4% for those who started CFDC ≤ 5 days of index culture, 19.4% for 6-20 days, and 31.3% for > 20 days. Crude 14-day and 28-day IHACM from index culture or starting CFDC had the same trend and were lower than overall IHACM (see Table 2).

**Figure:**
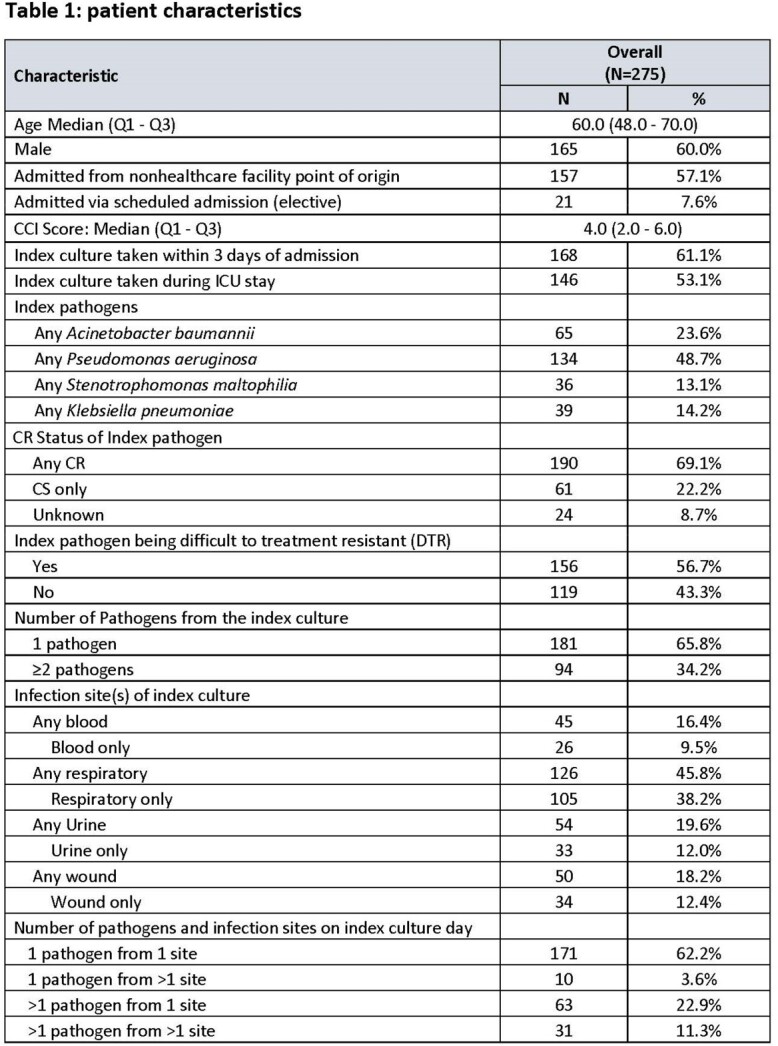

**Figure:**
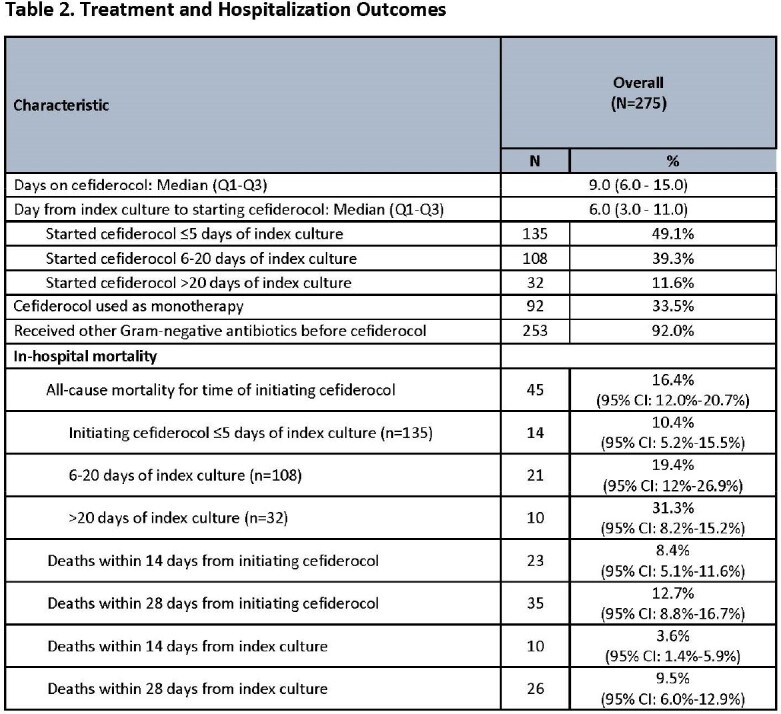

**Conclusion:**

The study demonstrated that CFDC is effective in treating patients with GN infections. Patients receiving CFDC earlier had lower IHACM than those receiving CFDC later but need further investigation.

**Disclosures:**

**Bin Cai, MD, PhD**, Shionogi Inc.: Shionogi employee **Christine M. Slover, PharmD**, Shionogi,INC: Employee **Jennifer D. copeland, MS**, Shionogi, Inc.: Employee (Medical/Micro) **Sean T. Nguyen, PharmD**, Shionogi: Employee|Shionogi, Inc: Employee

